# A Small Molecule–Drug Conjugate (SMDC) Consisting of a Modified Camptothecin Payload Linked to an α_V_ß_3_ Binder for the Treatment of Multiple Cancer Types

**DOI:** 10.3390/cancers14020391

**Published:** 2022-01-13

**Authors:** Hans-Georg Lerchen, Beatrix Stelte-Ludwig, Charlotte Kopitz, Melanie Heroult, Dmitry Zubov, Joerg Willuda, Thomas Schlange, Antje Kahnert, Harvey Wong, Raquel Izumi, Ahmed Hamdy

**Affiliations:** 1 Vincerx Pharma GmbH, 40789 Monheim am Rhein, Germany; beatrix.stelte-ludwig@vincerx.com; 2 Nuvisan Innovation Campus, 13353 Berlin, Germany; charlotte.kopitz@nuvisan.com; 3 Crop Science Division, Bayer AG, 65926 Frankfurt am Main, Germany; melanie.heroult@bayer.com; 4 Pharmaceuticals R&D, Bayer AG, 42096 Wuppertal, Germany; dmitry.zubov@bayer.com (D.Z.); thomas.schlange@boehringer-ingelheim.com (T.S.); antje.kahnert@bayer.com (A.K.); 5 Pharmaceuticals R&D, Bayer AG, 13353 Berlin, Germany; joerg.willuda@bayer.com; 6 Vincerx Pharma Inc., Palo Alto, CA 94306, USA; harvey.wong@vincerx.com (H.W.); raquel.izumi@vincerx.com (R.I.); ahmed.hamdy@vincerx.com (A.H.)

**Keywords:** α_V_β_3_ integrin, camptothecin payload, neutrophil elastase, small molecule–drug conjugate, SMDC, SN38 derivative, topoisomerase 1, tumor microenvironment, VIP126, VIP236

## Abstract

**Simple Summary:**

Many cancer drugs are cytotoxic, which means that they kill cancer cells effectively but are also toxic to normal cells. To overcome this problem, we designed a novel compound, VIP236, which consists of three components. The first part enables the drug to target and bind to a protein called ‘α_V_β_3_ integrin’ on the surface of cancer cells. The second part is a new cytotoxic drug, VIP126. The third component links the two parts together and can only be sliced by the enzyme neutrophil elastase. Neutrophil elastase is found in high quantities in tumors, ensuring that VIP126 is released near the cancer cells and less around normal cells. VIP126, when delivered as a component of VIP236, kills tumor cells in mouse cancer models without the toxicity seen with standard chemotherapy. VIP236 may be a new modality for targeting cancer cells directly, while reducing the harmful effects to normal tissue.

**Abstract:**

To improve tumor selectivity of cytotoxic agents, we designed VIP236, a small molecule–drug conjugate consisting of an α_V_β_3_ integrin binder linked to a modified camptothecin payload (VIP126), which is released by the enzyme neutrophil elastase (NE) in the tumor microenvironment (TME). The tumor targeting and pharmacokinetics of VIP236 were studied in tumor-bearing mice by in vivo near-infrared imaging and by analyzing tumor and plasma samples. The efficacy of VIP236 was investigated in a panel of cancer cell lines in vitro, and in MX-1, NCI-H69, and SW480 murine xenograft models. Imaging studies with the α_V_β_3_ binder demonstrated efficient tumor targeting. Administration of VIP126 via VIP236 resulted in a 10-fold improvement in the tumor/plasma ratio of VIP126 compared with VIP126 administered alone. Unlike SN38, VIP126 is not a substrate of P-gp and BCRP drug transporters. VIP236 presented strong cytotoxic activity in the presence of NE. VIP236 treatment resulted in tumor regressions and very good tolerability in all in vivo models tested. VIP236 represents a novel approach for delivering a potent cytotoxic agent by utilizing α_V_β_3_ as a targeting moiety and NE in the TME to release the VIP126 payload—designed for high permeability and low efflux—directly into the tumor stroma.

## 1. Introduction

Therapeutics designed to specifically target cancer cells by conjugating cytotoxic agents to selective tumor-targeting molecules are a promising strategy for drug development. Recent research interest has previously focused on antibody–drug conjugates (ADCs) that utilize antibodies to deliver potent cytotoxic agents [1,2,3]. In the past 10 years, altogether 12 ADCs have been approved for patient treatment, including Adcetris^®^ in 2011 [4,5]; Kadcyla^®^ in 2013 [6,7]; Besponsa^®^ in 2017 [8]; Mylotarg™ (re-approval in 2018) [9,10]; Polivy™^,®^ [11], Padcev^®^, and Enhertu^®^ in 2019 [12,13]; Trodelvy^®^ and Blenrep in 2020 [14]; and Zynlonta^®^ [15], Aidixi^®^ [16], and Tivdak^TM^ [17] in 2021.

**Figure 1:**
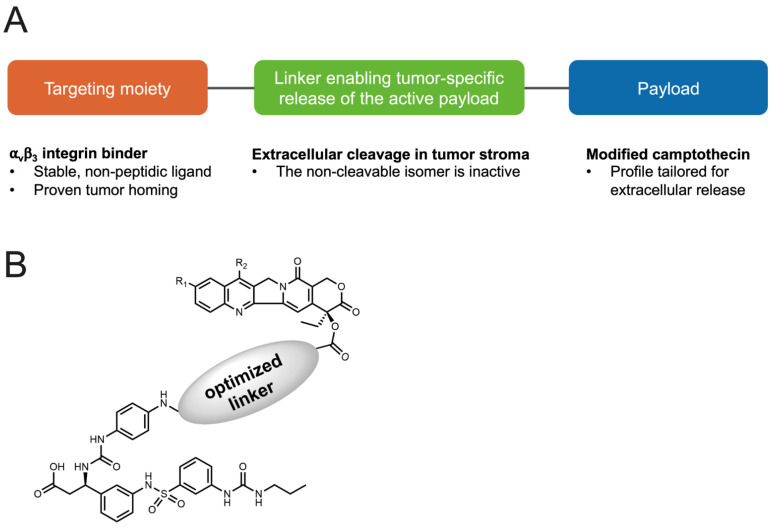
Structure of the small molecule–drug conjugate VIP236. (**A**) The small molecule–drug conjugate (SMDC) VIP236 targets α_V_β_3_ integrin, which is abundantly expressed on tumor cells. The VIP126 payload is specifically released by NE within the TME. (**B**) The generic chemical structure of VIP236.

ADCs, however, have several disadvantages related to molecular size, intracellular penetration, or pharmacokinetic issues, and current iterations still often induce severe toxicities listed as black box warnings on their prescribing information [18,19,20,21,22,23,24,25,26,27,28]. Small molecule–drug conjugates (SMDCs), which use biomarker-targeted small molecule compounds as the targeting moieties, provide a new perspective for targeted delivery and can address some of the issues related to ADCs. SMDCs are non-immunogenic by nature, have lower molecular weights to support good penetration into solid tumors, and their synthesis is more manageable and cost effective [29,30], making them a promising alternative to ADCs. Here, we describe VIP236, an SMDC consisting of an α_V_β_3_ integrin-targeting moiety and a neutrophil elastase (NE)-cleavable linker with a modified camptothecin (CPT), VIP126, as the cytotoxic payload (Figure 1).

The modular composition of the SMDC enabled us to optimize the components separately while considering opportunities for synergy. We reasoned that the success of the SMDC approach would be highly dependent on the cytotoxic payload employed. The CPT class of cytotoxic agents is potent with a well-understood mechanism-of-action [31]. Inhibition of topoisomerase I by related anti-cancer agents, such as irinotecan, topotecan, and belotecan, has been clinically validated but side effects still limit their clinical use. Furthermore, the failures of a large number of CPT derivatives in clinical development due to safety, rather than efficacy issues, suggest that targeted delivery of these compounds to tumors may increase their therapeutic indices. This has been demonstrated for recently approved ADCs with CPT payloads, such as trastuzumab deruxtecan (Enhertu^®^) [12] and sacituzumab govitecan (Trodelvy^®^) [14]. In addition, we have previously shown that the hydroxy lactone ring present in CPTs can be an appropriate linker attachment point with a beneficial impact on lactone ring stability, which is key for CPT activity [32,33]. VIP126, the modified CPT payload employed in VIP236, is a novel derivative of SN38, the clinically validated active metabolite of irinotecan (Camptosar^®^) [34]. It has been optimized for higher cell membrane permeability and significantly reduced efflux potential compared with SN38.

The α_V_β_3_ integrin-targeting moiety in VIP236 was selected based on the abundant expression of this cell surface protein on cancer cells and activated endothelial cells in a wide range of tumor types [35,36,37,38,39,40,41]. Previous experimental data suggested that the inhibition of α_V_β_3_ integrins could suppress tumor angiogenesis without affecting quiescent endothelial cells in normal tissues [42]. Therefore, the inhibition of integrins with multiple modalities, including antibodies and small molecules, has been considered a versatile approach to inhibit tumor angiogenesis. However, despite promising preclinical results demonstrating that the inhibition of integrins has therapeutic potential, clinical trials with integrin inhibitors have repeatedly failed to demonstrate benefits in cancer patients [43]. Nevertheless, imaging and tissue analyses have demonstrated that the studied integrin inhibitors, for example cilengtide and volociximab, reach their targets [43].

The binding of integrins, such as α_V_β_3_ to arginine-glycine-aspartic acid (RGD) sequences present on many extracellular matrix proteins, has been well characterized [44,45,46,47], and the use of synthetic RGD peptides to target α_V_β_3_ integrins presents an attractive mechanism for selective delivery of cytotoxic agents into the tumor microenvironment (TME). Indeed, α_V_β_3_ targeting conjugates and nanoparticles have been investigated to deliver chemotherapy compounds [48,49,50], gene therapy [51], and siRNA [52] to tumor blood vessels to block tumor growth and metastasis.

Extracellular enzymatic cleavage of the VIP236 conjugate is essential for the release of the active agent VIP126. The design of VIP236 with a cleavage site for NE, a highly specific protease enriched in the TME of many malignant tumors [53], enabled us to further restrict the delivery of the cytotoxic payload. As for α_V_β_3_ integrins, neutrophil infiltration and elastase expression in tumors have been shown to correlate with their metastatic potential and poor prognosis [54]. Therefore, we hypothesized that the α_V_β_3_ integrin-targeting moiety combined with the NE-specific linker in VIP236 could be ideally suited for restricting delivery of the novel cytotoxic payload VIP126 into the TME.

Herein, we describe the pharmacokinetic characteristics and in vitro and in vivo efficacy of VIP236—a novel SMDC consisting of an α_V_β_3_ integrin binder and an NE-cleavable linker connected to the cytotoxic payload VIP126.

## 2. Materials and Methods

### 2.1. Cell Lines

Caco-2 human colon adenocarcinoma, NCI-H1975 human non-small cell lung cancer (NSCLC), 786-O human RCC, HT-29 human CRC, LoVo human CRC, SW480 human CRC, NCI-H292 human pulmonary mucoepidermoid carcinoma, NCI-H69 human SCLC, 4T1 mouse breast cancer, and MX-1 human triple negative breast cancer cells were acquired from ATCC and cultured according to the provider’s instructions. LLC-PK1 porcine renal epithelial cells overexpressing human P-gp (L-MDR1 cells) were obtained from Prof. Schinkel at Netherlands Cancer Institute (Amsterdam, The Netherlands). The recombinant NCI-H1975-P-gp and NCI-H1975-BCRP NSCLC cell lines were generated by the Natural and Medical Sciences Institute (NMI; University of Tuebingen, Tuebingen, Germany) by lentiviral transduction using the plasmids pGT414_Hyg_PGP and pGT414_Hyg_BCRP. The cancer cell lines were authenticated using short tandem repeat DNA fingerprinting at DSMZ and subjected frequently to mycoplasma testing.

### 2.2. Compounds

The SMDC VIP236, consisting of the α_V_β_3_ binder, the modified CPT payload VIP126, and the NE-cleavable linker were synthesized at Bayer AG. The dye conjugate BAY810, consisting of the α_V_β_3_ binder BAY812 coupled to IRDye^®^ 800CW (from IRDye^®^ 800RS NHS Ester; LI-COR Bioscience, Bad Homburg, Germany), and BAY813, a non-binding control conjugate of IRDye^®^ 800CW, were also synthesized at Bayer AG. Topotecan (Hycamtin^®^) was purchased from GlaxoSmithKline (GSK, Brentford, UK). Irinotecan (camptothecin-11) was purchased from Sanofi-Aventis (Paris, France). SN38 was synthesized at Bayer AG. Doxorubicin, cisplatin, and 5-FU were purchased from Sigma-Aldrich (Darmstadt, Germany).

### 2.3. Caco-2 and P-gp-Expressing LLC-PK1 Cell Permeability Assays

The cell permeability of the VIP126 and SN38 payloads was investigated with in vitro flux assays using Caco-2 cells [55] or P-gp-overexpressing LLC-PK1 porcine renal epithelial cells (L-MDRl cells) [56]. VIP126 and SN38 were dissolved in a HEPES buffer and applied (in triplicate) to the cells either apically (A) or basolaterally (B) at a final concentration of 2 μM. Before and after incubation for 2 h at 37 °C, samples were taken from both compartments and analyzed by LC-MS/MS. The apparent permeability coefficient (*P*_app_) was calculated for both the apical to basolateral (A → B) and the basolateral to apical (B → A) direction as described by Schwab et al. [57].

### 2.4. Cytotoxicity of the VIP126 and SN38 Payloads in NCI-H1975 Parental and Transporter-Expressing Mutant Cells

The in vitro cytotoxicity of the VIP126 and SN38 payloads was investigated in parental NCI-H1975 and in transfected NCI-H1975-P-gp and NCI-H1975-BCRP NSCLC cells. Cells (6 × 10^3^ cells/well) were plated in RPMI-1640 medium supplemented with 2% fetal calf serum (FCS) minimal media (MM) in 96-well plates and allowed to adhere at 37 °C/5% CO_2_ for 24 h. Then, the test compounds VIP126 and SN38 were added at concentrations of 1000–250–62.5–15–4–1–0.25 nM in MM (no elastase, final dimethyl sulfoxide (DMSO) concentration of 0.1%) in triplicates. The cells were incubated at 37 °C/5% CO_2_ for 72 h. Then, Alamar Blue (#DAL1100, Invitrogen, Waltham, MA, USA) was added at a 1:1 ratio, the cells were incubated at 37 °C/5% CO_2_ for 4 h, and fluorescence (530 nm/590 nm) was measured with a VICTOR X2 Multilabel Reader (PerkinElmer, Waltham, MA, USA). The fluorescent signal was directly proportional to the number of viable cells. The half-maximal growth inhibition (IC_50_) was determined from the dose–response curves using the GraphPad Prism software.

### 2.5. In Vitro Proliferation Assay

The antiproliferative activity of VIP236 and VIP126 was evaluated in a panel of cancer cell lines using the MTT Cell Proliferation Assay (ATCC). Briefly, cells (1.5–2.5 × 10^3^ cells/well) were plated in their appropriate growth medium in 96-well plates, allowed to adhere at 37 °C/5% CO_2_ for 24 h, and then the test compounds were added at concentrations of 1 × 10^−5^–1 × 10^−13^ M in triplicates. Two identical sets of samples were prepared; the first set was treated with the test compound alone and the second set was treated with the test compound and 10 nM NE (Sigma-Aldrich). Cells were incubated at 37 °C/5% CO_2_ for 72 h, and the proliferation was determined using the MTT assay according to the manufacturer’s instructions. The proliferation of untreated, but otherwise identically handled, cells was defined as 100%. The half-maximal growth inhibition (IC_50_) was determined from the dose–response curves using the GraphPad Prism software.

### 2.6. Tumor Homing of the α_V_β_3_ Binder in 786-O RCC Tumor-Bearing Mice

All animal experiments were conducted in accordance with the German Animal Welfare Law and approved by local authorities. To study the tumor homing of the α_V_β_3_ binder, male BALB/c nude mice (11 weeks; Taconic M&B A/S, Lille Skensved, Denmark) were inoculated subcutaneously (s.c.) with 2 × 10^6^ 786-O RCC cells suspended in phosphate-buffered saline (PBS). The mice were fed with chlorophyll-free chow (Harlan, Horst, The Netherlands) 7 days prior to imaging to reduce the background fluorescence. When the 786-O tumors reached an average tumor size of 0.7 cm in diameter, the mice (*n* = 3 mice/group) were injected with 20 nmol of BAY810 (1.35 mg/kg), consisting of the α_V_β_3_ binder coupled to IRDye^®^ 800CW (LI-COR Bioscience, Bad Homburg, Germany), according to the manufacturer’s instructions, or BAY813 (1.35 mg/kg), a non-binding control conjugate of IRDye^®^ 800CW. As a negative control, one mouse was injected with IRDye^®^ 800CW carboxylate (0.84 mg/kg) in a sterile 0.9% NaCl aqueous solution (Sigma-Aldrich). Tumor accumulation of the IRDye^®^ conjugates in mice was determined 8, 24, and 48 h post administration using the LI-COR Pearl^®^ imaging system (LI-COR Bioscience, Bad Homburg, Germany).

### 2.7. Pharmacokinetics of VIP236 and VIP126 in Plasma and Tumor

The pharmacokinetic properties of VIP236 and VIP126 in plasma and tumor were estimated in female NMRI nu/nu xenograft mice (Taconic M&B A/S) bearing 786-O human RCC tumors. The mice were treated intravenously (i.v.) via the tail vein with a single dose of VIP236 (4 mg/kg) or VIP126 (1 mg/kg). The doses of VIP236 and VIP126 were chosen so that an equimolar amount of VIP126 was administered regardless of whether it was delivered utilizing VIP236 or delivered directly. Plasma and tumor samples were collected at 2 min, 10 min, 30 min, 1 h, 2 h, 4 h, 7 h, and 24 h after treatment (*n* = 2 mice/group at each time point), and the concentrations of VIP236 and VIP126 were measured by LC-MS/MS. Pharmacokinetic analysis of VIP236 and VIP126 was performed using the mean concentration–time profiles. All pharmacokinetic parameters were calculated by non-compartmental methods, as previously described [58].

### 2.8. In Vivo Antitumor Efficacy of VIP236

The in vivo antitumor efficacy of VIP236 was evaluated in the 786-O (human RCC), MX-1 (human TNBC), NCI-H69 (human SCLC), and SW480 (human colorectal cancer) xenograft models.

VIP236 was formulated in PBS. Irinotecan, doxorubicin, topotecan, cisplatin, and 5-FU were diluted in 0.9% NaCl. In all in vivo antitumor efficacy studies, the vehicle control group was treated with the VIP236 formulation buffer. 

In the 786-O efficacy study, immunocompromised female NMRI nu/nu mice (8–10 weeks, 21–22 g, Taconic M&B A/S) were inoculated s.c. with 2 × 10^6^ 786-O cells suspended in 100% Matrigel^®^. The mice were randomized into control and treatment groups (*n* = 8) when the 786-O tumors reached an average size of 25 mm^2^. The 786-O tumor-bearing mice were treated with vehicle or VIP236 (36 mg/kg, 3 on/4 off, i.v.).

In the MX-1 efficacy study, the in vivo-grown tumor fragments (2 mm × 2 mm × 2 mm) were implanted s.c. in immunocompromised female NMRI nu/nu mice (8–10 weeks, 18–21 g, Taconic M&B A/S). The mice were randomized into control and treatment groups (*n* = 8) when the MX-1 tumors reached an average size of 25 mm^2^. The MX-1 tumor-bearing mice were treated with vehicle, VIP236 at 23, 36, or 40 mg/kg (3 days on/4 days off (3 on/4 off), i.v.), irinotecan at 15 mg/kg (4 on/3 off, i.v.) or 30 mg/kg (Q2/3Dx9, i.v.), or doxorubicin at 10 mg/kg (Q14Dx2, i.v.).

In the NCI-H69 efficacy study, immunocompromised female NMRI nu/nu mice (8–10 weeks, 18–21 g, Taconic M&B A/S) were inoculated s.c. with 3 × 10^6^ NCI-H69 cells suspended in 50% Matrigel^®^/50% medium. The mice were randomized into control and treatment groups (*n* = 8) when the NCI-H69 tumors reached an average size of 25 mm^2^. The NCI-H69 tumor-bearing mice were treated with vehicle, VIP236 (40 mg/kg, 3 on/4 off, i.v.), topotecan (0.5 mg/kg, 7 on/7 off, i.v.), or cisplatin (3 mg/kg, Q3Dx14, intraperitoneally [i.p.]).

In the SW480 efficacy study, immunocompromised female NMRI nu/nu mice (8–10 weeks, 18–21 g, Taconic M&B A/S) were inoculated subcutaneously with 3 × 10^6^ SW480 cells suspended in 50% Matrigel^®^/50% medium. The mice were randomized into control and treatment groups (*n* = 8) when the SW480 tumors reached an average size of 25 mm^2^. The SW480 tumor-bearing mice were treated with vehicle, VIP236 at 23, 36, or 40 mg/kg (3 on/4 off, i.v.), irinotecan at 15 mg/kg (4 on/3 off, i.v.) or 30 mg/kg (Q2/3Dx9, i.v.), or 5-FU at 100 mg/kg (Q7D, i.p.).

Subcutaneous tumor growth was monitored by measuring tumor area (length × width) using a caliper. Animal body weight was monitored as an indicator of treatment-related toxicity. Measurement of tumor area and body weight was performed at least twice weekly. Individual animals were sacrificed when showing >20% body weight loss or when tumors reached a maximum area of ~200 mm^2^ unless ulceration or other predefined humane endpoints were met. At study termination, the animals were sacrificed by cervical dislocation under CO_2_ anesthesia. T/C (treatment/control) ratios were calculated using the final mean tumor areas at study or control group termination. Treatment responses were defined on the last day of the vehicle group (Day 38 for 786-O, Day 86 for MX-1, Day 31 for NCI-H69, and Day 24 for SW480) using the RECIST criteria [59]. Progressive disease (PD) was defined as a greater than 20% increase in tumor size. Partial response (PR) was defined as a greater than 30% reduction in tumor size. Complete response (CR) was defined as an absence of any palpable tumor mass. No tumor growth or a slight reduction (<30%) or small increase (<20%) in tumor size was defined as stable disease (SD).

### 2.9. Statistical Analyses

Statistical analyses were performed using the GraphPad Prism software (version 9). Tumor area data were analyzed using one-way ANOVA followed by Dunnett’s test or paired *t*-tests. *p* values < 0.05 were considered significant.

## 3. Results

### 3.1. The α_V_β_3_ Integrin-Targeting Moiety Mediates Tumor Homing

**Figure 2:**
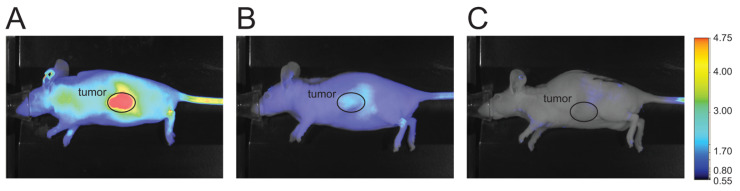
The α_V_β_3_ integrin binder shows tumor homing in 786-O RCC tumor-bearing mice. Tumor homing of (**A**) the IR800-coupled α_V_β_3_ binder conjugate, (**B**) the non-binding control conjugate, or the (**C**) carboxylate dye control in 786-O tumor-bearing mice as detected by near-infrared imaging. The color scale shows arbitrary near-infrared fluorescence units as displayed by the Pearl^®^ Imager software. A representative image from each treatment group is shown (*n* = 3).

First, we investigated the tumor homing ability of the α_V_β_3_ integrin binder in 786-O human renal cell carcinoma (RCC) tumor-bearing mice via conjugation to a fluorescent IR800 dye using in vivo near-infrared imaging. We used an i.v. dose of 1.35 mg/kg for the IR800-coupled α_V_β_3_ integrin binder and the non-binding IR800-coupled control conjugate or 0.84 mg/kg for the carboxylate dye negative control (equaling 20 nmol) per mouse, and the mice were imaged 8, 24, and 48 h post administration. The IR800-coupled α_V_β_3_ integrin binder showed strong tumor homing in 786-O RCC tumor-bearing mice (Figure 2A), whereas no tumor-specific accumulation was detected with the non-binding IR800-coupled control conjugate (Figure 2B). Overall signal intensity in mice injected with the carboxylate negative control dye was low at all three time points, indicating that background near-infrared fluorescence due to unspecific dye binding was negligible (representative image shown for the 48-h time point, Figure 2C). Injection of the non-binding control resulted in a non-tumor specific signal mainly located in the skin and kidneys, suggesting renal clearance of the dye conjugate. While the overall signal intensity weakened over time when using the α_V_β_3_-binding conjugate, the relative near-infrared fluorescence signal showed an up to 42-fold increase in the tumor compared with other organs, including the kidneys, lung, spleen, and liver (data not shown).

### 3.2. The Modified CPT Payload VIP126 Shows an Improved In Vitro Profile

Transporter pumps, such as the P-glycoprotein (P-gp) efflux pump expressed on cancer cells, are known to contribute to resistance to chemotherapy treatment [60]. The cytotoxic payload VIP126 is released extracellularly from the SMDC, and therefore, cellular permeability and the potential to resist efflux transporter pumps are important aspects in the selection of the antineoplastic agent to be used as the payload. SN38 is a clinically validated CPT derivative that has demonstrated high performance as the active metabolite of the small molecule irinotecan (Camptosar^®^) as well as the payload of the ADC sacituzumab govitecan (Trodelvy^®^) [61]. Here, we compared the permeability and efflux ratio of VIP126 to SN38 using Caco-2 cells and human P-gp-expressing LLC-PK1 cells. VIP126 showed 20-times higher permeability compared with SN38, and its efflux ratio was approximately 1 in Caco-2 and human P-gp-expressing LLC-PK1 cells, suggesting that the compound was not transported out of the cells (Table 1). This indicated that, unlike SN38, VIP126 was not a substrate for P-gp-mediated efflux transportation. Furthermore, VIP126 demonstrated comparable cytotoxicity in parental NCI-H1975 cells and in P-gp- or breast cancer resistance protein (BCRP) efflux transporter-expressing mutants. In contrast, SN38 showed lower cytotoxicity in the efflux transporter-expressing NCI-H1975 mutants, demonstrated by 3–11 times higher half-maximal growth inhibition (IC_50_) values compared with the parental NCI-H1975 cells (Table 2 and Supplementary Figure S1).

### 3.3. In Vitro Cytotoxic Activity of VIP236 against Tumor Cell Lines Is NE-Dependent

VIP236 is an SMDC consisting of the α_V_β_3_ binder and the VIP126 payload, which is connected via a linker designed to be selectively cleaved by NE. Since tumor cell lines do not express NE, we evaluated the in vitro cytotoxic activity of the VIP236 conjugate in the presence or absence of NE in a panel of cancer cell lines. VIP236 showed weak antiproliferative activity in the absence of NE, with IC_50_ values in the two to three-digit nanomolar range. When NE was added to the culture medium to simulate the TME, the cytotoxic activity of VIP236 increased to levels comparable to the VIP126 payload alone, reaching single-digit nanomolar IC_50_ values (Table 3 and Supplementary Figure S2).

### 3.4. VIP236 Has Low Clearance and Results in Reduced Systemic Exposure of the Payload

**Figure 3:**
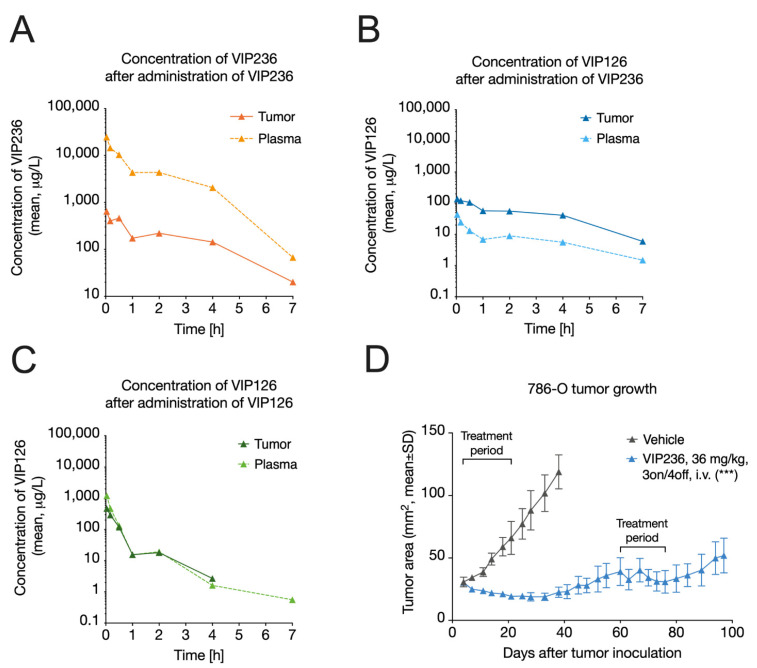
The higher tumor-to-plasma ratio of VIP126 after VIP236 administration results in marked anti-tumor efficacy in vivo. Concentration of VIP236 and VIP126 in tumor and plasma following the administration of (**A**,**B**) the VIP236 conjugate or (**C**) the VIP126 payload directly. 786-O tumor-bearing mice (*n* = 2 mice/group at each timepoint) were treated i.v. with a single dose of VIP236 (4 mg/kg) or VIP126 (1 mg/kg, via tail vein). The doses of VIP236 and VIP126 were chosen so that an equimolar amount of VIP126 was administered regardless of whether it was delivered utilizing VIP236 or delivered directly. (**D**) Growth curves of 786-O tumors in female NMRI nu/nu mice (*n* = 8/group) treated with vehicle or VIP236 (36 mg/kg, 3 on/4 off, i.v.). Statistical analyses were performed using paired *t*-tests on Day 38 in (**D**). *** *p* < 0.001 compared with vehicle.

The plasma and tumor concentration–time profiles of the VIP236 conjugate and the VIP126 payload following i.v. administration of equimolar doses of VIP126 delivered as the VIP236 conjugate or directly were determined in 786-O RCC tumor-bearing mice. First, we determined the pharmacokinetic properties of the VIP236 conjugate and the VIP126 payload upon their direct i.v. administration in 786-O tumor-bearing mice (Figure 3A,C; Table 4). Clearance (CL) was low for VIP236 and moderate for VIP126. The volume of distribution at steady-state (V_ss_) was low for VIP236 and the approximated plasma volume, suggesting limited distribution outside of plasma [62]. In contrast, for VIP126, the V_ss_ was moderate, being 22-fold higher compared with VIP236, suggestive of higher penetration into cells. The half-life (≤1 h) was short for both compounds. Finally, the delivery of VIP126 via VIP236 resulted in reduced systemic exposure of VIP126, as demonstrated by a ~6-fold lower area under the curve value (AUC_plasma_) compared with direct VIP126 delivery. The reduced systemic exposure is anticipated to reduce potential adverse effects (Table 5).

### 3.5. VIP236 Results in a Higher Tumor-to-Plasma Ratio of the VIP126 Payload and Leads to Anti-Tumor Efficacy In Vivo

Delivery of the VIP126 payload via VIP236 conjugate administration resulted in altered VIP126 tumor exposure when compared with direct administration. Despite having lower VIP126 plasma exposure with VIP236 administration, VIP126 tumor exposure (AUC_tumor_) was ~72% higher compared with direct VIP126 administration (Figure 3B,C; Table 4). Notably, the VIP126 tumor-to-plasma ratio (AUC_tumor_/AUC_plasma_) with VIP236 conjugate administration was 6 (Figure 3B; Table 5), whereas a ratio of 0.6 was observed when an equimolar dose of VIP126 was administered directly (Figure 3C; Table 5). This indicated a 10-fold improvement in the tumor-to-plasma ratio, highlighting a potential therapeutic advantage. Indeed, in the 786-O RCC xenograft model, marked anti-tumor efficacy was observed upon treatment with VIP236 at 36 mg/kg (3 on/4 off, i.v.) with a T/C value 0.19 (*p* < 0.001) on Day 38, when the vehicle group was sacrificed (Figure 3D). Furthermore, 3/8 and 5/8 of the VIP236-treated mice had partial responses (PRs) and stable diseases (SDs), respectively. Importantly, the tumors also responded to the second treatment period between Days 60 and 80.

### 3.6. VIP236 Shows High Antitumor Efficacy and Good Tolerability In Vivo

Finally, we extended the evaluation of the in vivo antitumor efficacy and tolerability of the VIP236 conjugate by comparing it to commonly utilized anticancer compounds using the MX-1 triple-negative breast cancer (TNBC), NCI-H69 small cell lung cancer (SCLC), and SW480 colorectal cancer (CRC) xenograft mouse models.

**Figure 4:**
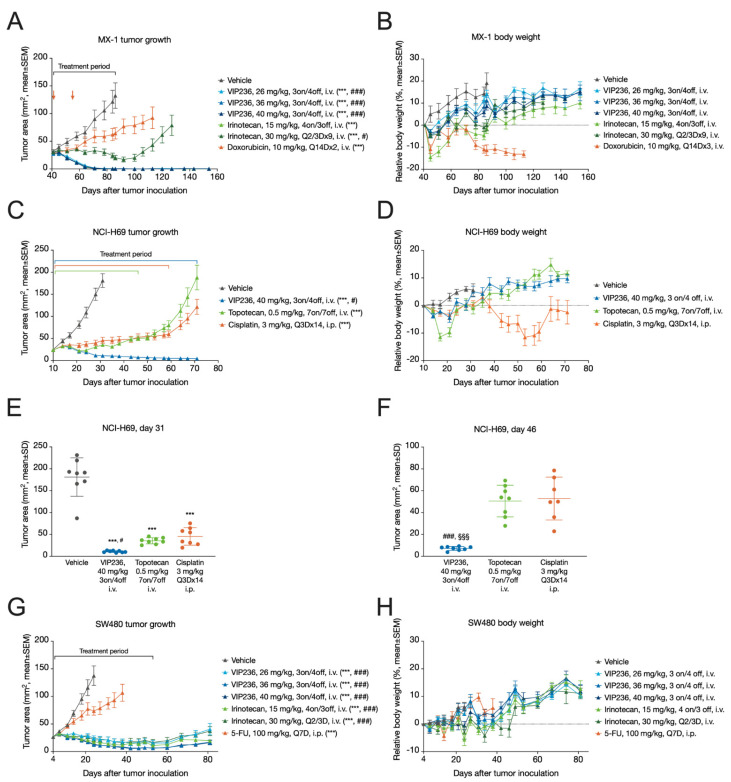
Antitumor efficacy of VIP236 and reference compounds in mouse xenograft models. (**A**) Growth curves of MX-1 TNBC tumors in female NMRI nu/nu mice (*n* = 8/group) treated with vehicle, VIP236 (23, 36, or 40 mg/kg, 3 on/4 off, i.v.), irinotecan (15 mg/kg, 4 on/3 off, i.v. or 30 mg/kg, Q2/3Dx9, i.v.), or doxorubicin (10 mg/kg, Q14Dx2, i.v.). (**B**) Relative body weight change of the MX-1 tumor-bearing mice treated as described in (**A**). (**C**) Growth curves of NCI-H69 SCLC tumors in female NMRI nu/nu mice (*n* = 8/group) treated with vehicle, VIP236 (40 mg/kg, 3 on/4 off, i.v.), topotecan (0.5 mg/kg, 7 on/7 off, i.v.), or cisplatin (3 mg/kg, Q3Dx14, i.p.). (**D**) Relative body weight change of the NCI-H69 tumor-bearing mice treated as described in (**C**). (**E**) Areas of the NCI-H69 tumors described in (**C**) on the last day of the vehicle group (Day 31). (**F**) Areas of the NCI-H69 tumors described in (**C**) on the last day of the topotecan treatment group (Day 46). (**G**) Growth curves of SW480 tumors in female NMRI nu/nu mice (*n* = 8/group) treated with vehicle, VIP236 (23, 36, or 40 mg/kg, 3 on/4 off, i.v.), irinotecan (15 mg/kg, 4 on/3 off, i.v.; or 30 mg/kg, Q2/3Dx9, i.v.), or 5-FU (100 mg/kg, Q7D, i.p.). (**H**) Relative body weight change of the SW480 tumor-bearing mice treated as described in (**G**). Statistical analyses were performed using one-way ANOVA followed by Dunnett’s test on Day 86 in (**A**), Days 31 and 46 in (**C**), or Day 24 in (**G**). *** *p* < 0.001 compared with vehicle; ^#^ *p* < 0.05 compared with doxorubicin (**A**) or cisplatin (**C**,**E**); ^###^ *p* < 0.001 compared with doxorubicin in (**A**), cisplatin in (**F**), or 5-FU in (**G**). ^§§§^ *p* < 0.001 compared with topotecan in (**F**).

In the MX-1 TNBC model, complete tumor responses (CRs) were observed in >80% of mice across all three VIP236 doses (26–40 mg/kg, 3 days on/4 days off, i.v.; Table 6). No tumor regrowth was observed in any of the VIP236 treatment groups 68 days after the last treatment on Day 86 (Figure 4A). Similar results were observed with irinotecan at 15 mg/kg (4 on/3 off, i.v.). In contrast, irinotecan at 30 mg/kg (Q2/3Dx9, i.v.) resulted in stable disease, but clear tumor regrowth was observed after the treatment was stopped. Furthermore, the irinotecan treatment resulted in a relative body weight loss of >10%, which required treatment holidays. Doxorubicin (10 mg/kg, Q14Dx2, i.v.) was the least effective agent in this TNBC xenograft model (Figure 4A) and had the most toxicity with a relative body weight loss of >15% (Figure 4B). VIP236 was efficacious when compared with placebo (*p* < 0.001) and with doxorubicin (*p* < 0.001) with less toxicity (Figure 4A,B).

In the NCI-H69 SCLC model, treatment with VIP236 at 40 mg/kg (3 on/4 off, i.v.) showed marked antitumor efficacy with a T/C of 0.06 and partial responses (PRs) in 8/8 mice (Figure 4C; Table 6) and was well tolerated (Figure 4D). The VIP236 conjugate was more efficacious than cisplatin (3 mg/kg, Q3Dx14, i.p.) on Day 31, when the vehicle group was sacrificed (*p* = 0.022) (Figure 4E). On Day 46, when the topotecan group (0.5 mg/kg, 7 on/7 off, i.v.) was sacrificed, VIP236 was more efficacious than cisplatin and topotecan with *p*-values < 0.001 for both treatments (Figure 4F). In contrast to VIP236, topotecan and cisplatin treatments resulted in a relative body weight loss of >10%. In the cisplatin treatment group, one animal died on Day 46.

In the SW480 CRC model, VIP236 treatment resulted in clear tumor growth inhibition at all three doses, similar to irinotecan (Figure 4G,H; Table 6). Treatment with VIP236 at 40 mg/kg (3 on/4 off, i.v.) resulted in a T/C of 0.10 and PRs in 8/8 mice. After treatment cessation, moderate tumor regrowth was observed in all VIP236- and irinotecan-treated mice. VIP236 and irinotecan were significantly (*p* < 0.001) more efficacious than 5-fluorouracil (5-FU), a chemotherapy commonly used to treat CRC.

## 4. Discussion

We developed a potent modified CPT payload, VIP126, to be selectively delivered to cancer cells by an SMDC targeting α_V_β_3_ integrin. In the TME, the payload is cleaved by NE and enters cancer cells to induce DNA damage and cell killing [63]. Many tumor cells express elevated levels of the ATP-binding cassette (ABC) transporter family members, such as P-gp or BCRP, and efflux through these transporters is one of the most widely recognized mechanisms of multidrug resistance [64]. In contrast with SN38, the VIP126 payload retained its cytotoxic activity in P-gp or BCRP transporter-transfected cells. This indicates that VIP126 is not a substrate of such transporter proteins and that it could exhibit an improved activity profile even in tumors that have progressed or relapsed after one or more lines of chemotherapy. 

The integrin target was selected because of its extensive characterization in normal and tumor tissues. Integrin α_V_β_3_ is most abundantly expressed on angiogenic endothelial cells in remodeling and pathological tissues [65]. In the TME, α_V_β_3_ integrins play critical roles in tumor progression, resistance to cytotoxic therapy, metastasis, and the recruitment of immune and inflammatory cells (reviewed in [43]). They have been implicated in cancer growth and invasion in a wide variety of solid tumors, including melanoma, pancreatic, prostatic, breast, ovarian, and cervical cancers [35,36,37,38,39,40,41]. The expression of α_V_ and β_3_ integrins has been associated with poor prognosis in cancers such as colorectal cancer [66,67] and melanoma [68]. In experimental studies, the inhibition of α_V_β_3_ integrins by antibodies, Arg–Gly–Asp-based cyclic peptides, or non-peptidic mimetics has been shown to suppress tumor angiogenesis without affecting quiescent endothelial cells [42], raising the expectation that α_V_β_3_ integrin inhibition might be a valuable anti-cancer strategy (reviewed in [69]). 

Although the inhibition of integrins has long been considered a viable approach to inhibit tumor angiogenesis and growth, this concept has not been successful in the clinic. Anti-α_V_β_3_ inhibitory antibodies, based on a humanized form of the mouse monoclonal LM609 (etaracizumab; abegrin; vitaxin), were tested and well tolerated [70,71], as were two pan-α_V_ integrin antibodies, abituzumab [72] and intetumumab [73]. In addition, the cyclic peptide cilengitide, which specifically inhibits α_V_β_3_ and α_V_β_5_ integrins, performed well in preclinical studies [74,75] and progressed to Phase 3 trials for cancer indications, including glioblastoma [76]. However, despite compelling preclinical results demonstrating the therapeutic potential of integrin inhibition, clinical trials repeatedly failed to demonstrate therapeutic benefits in cancer patients [73,77,78,79,80]. Furthermore, low concentrations of cilengitide were found to induce, rather than inhibit, α_V_β_3_ signaling, resulting in stimulated angiogenesis [81]. The failure of cilengitide and other α_V_β_3_-targeting molecules as anti-angiogenic drugs may be due to limitations intrinsic to all inhibitory approaches, in particular adaptive resistance [43]. Functional redundancy, promiscuity, and compensation typical of integrins [82] may be the reasons why these integrin inhibitors were well tolerated, but at the same time had limited therapeutic benefit. Nevertheless, imaging and tissue analyses indicated that small molecules, such as cilengitide, reach their targets [43]. 

Our approach employs a selective integrin α_V_β_3_ small molecule binder to focally concentrate a chemotherapeutic warhead to kill tumor cells and angiogenic endothelial cells. This approach does not depend on the inhibition of integrin signaling for its therapeutic effect. Using small molecules to target integrins for the delivery of cytotoxic payloads is a relatively new and untested strategy, which has not yet been advanced to clinical trials [50]. Previous attempts included ADCs such as IMGN-388, in which intetumumab was bound to the maytansinoid cytotoxic agent DM4. IMGN-388 was well tolerated in a Phase 1 trial [83]. However, there were no responses, even though some of the patients’ tumors had high integrin expression. There were several cases of disease stabilization as well as a correlation between integrin expression and dose to clinical benefit, but patient numbers were too small to draw definitive conclusions. In alternative strategies, α_V_β_3_ integrin-targeting nanoparticles were used with limited success to deliver chemotherapeutic compounds to tumors [48,49]. VIP236 extends and improves upon the integrin-targeted cytotoxic payload concept in several important ways.

VIP236 is an SMDC rather than an ADC, which affords certain advantages. SMDCs are less immunogenic and easier to synthesize [29]. For VIP236, we employed a peptidomimetic α_V_β_3_ ligand that demonstrated efficient tumor homing when conjugated to a fluorescent dye.

To further enhance the targeting specificity of the VIP236 SMDC, we engineered an NE cleavage site into the linker peptide that connects the targeting moiety to the payload and enables the release of the payload without the formation of intermediates. The cleavage site was optimized for effective and specific cleavage by NE in the TME and it is stable in blood plasma, which minimizes systemic payload release. This restricts the drug release to the extracellular regions of the tumor where high concentrations of the enzyme occur. NE is a member of a family of serine proteases that degrade elastin and other extracellular matrix proteins [84]. It is expressed by myeloid cells, including neutrophils and macrophages. NE expression and activity are upregulated in the microenvironment of numerous cancers, where the enzyme contributes to cancer progression by enhancing invasion and metastasis [84,85]. NE expression can also be found in tumor extracts originating from xenograft models [54]. Like α_V_β_3_ integrin, NE expression is also correlated with poor prognosis [54].

VIP236 exhibited strong biologic activity in in vitro and in vivo experiments. In cultured cancer cells, the compound showed weak antiproliferative activity in the absence of NE, with IC_50_ values in the mid-nanomolar range. When NE was added to the culture medium to simulate the TME, the antiproliferative activity of VIP236 increased to levels comparable to the VIP126 payload alone, with single-digit nanomolar IC_50_ values. This indicates that VIP236 functions as an antiproliferative compound only upon specific cleavage by a protease prevalent in the TME. The tumor-to-plasma ratios of the payload VIP126 were 10-fold enhanced when delivered as a conjugate versus direct administration. Finally, in vivo treatment with VIP236 resulted in tumor regression in all xenograft models tested (RCC, TNBC, SCLC, and CRC), with good tolerability. 

The SMDC VIP236 benefits from the synergy resulting from its modular composition. Tumor homing by α_V_β_3_ integrin targeting combined with payload release by NE in the TME focus the cytotoxic activity to the tumor. This TME targeting, along with the high conjugate stability in plasma and the release of VIP126 at its site of action, further supports the broadening of the therapeutic window. Moreover, the beneficial properties of VIP126 (i.e., high cellular permeability and low efflux ratio) present a promising approach to overcome common tumor resistance mechanisms. These findings support further development of VIP236 as a potential treatment option for advanced or invasive cancers. 

## 5. Conclusions

A novel SMDC VIP236 was developed and optimized to bind to α_V_β_3_ integrin on cancer cells and activated endothelial cells and to release its modified CPT payload VIP126 when selectively cleaved by NE in the TME. The α_V_β_3_ integrin binder demonstrated efficient tumor homing in vivo. VIP126 is a novel, modified SN38 derivative with higher cellular permeability and significantly reduced efflux potential. In contrast to SN38, VIP126 also retained its cytotoxic activity in transporter-expressing mutant cell lines. A 10-fold higher tumor-to-plasma ratio of VIP126 was shown after conjugate administration compared with i.v. administration of the payload alone. Treatment with VIP236 resulted in tumor regressions in all tested xenograft models in vivo (RCC, TNBC, SCLC, and CRC), with good tolerability.

## Figures and Tables

**Table 1:** Permeability and efflux ratio of the VIP126 and SN38 payloads measured in flux assays with human P-gp-expressing LLC-PK1 cells and Caco-2 cells. The values represent the mean value of at least two independent assays performed in triplicate (*n* = 3).

Cell Line	Payload	Permeability A → B [nm/s]	Efflux Ratio
P-gp-expressing LLC-PK1	VIP126	196	0.6
	SN38	10	16
Caco-2	VIP126	171	1
	SN38	8	36

**Table 2:** Cytotoxic activity (IC_50_) of VIP126 and SN38 in NCI-H1975 parental and efflux transporter-expressing mutant cells. The values represent the mean value of at least two independent assays performed in triplicate (*n* = 3).

Compound	IC_50_ [nM]		
	NCI-H1975	NCI-H1975-P-gp	NCI-H1975-BCRP
VIP126	19	34	27
SN38	45	141	512

IC_50_, half-maximal inhibitory concentration; VIP126, modified camptothecin payload.

**Table 3:** Cytotoxic activity (IC_50_) of VIP236 in the presence or absence of 10 nM NE in a panel of cancer cell lines. The values represent the mean value of at least two independent assays performed in triplicate (*n* = 3).

Cancer Cell Line		IC_50_ (nM)		
	Cancer Type	VIP236 without NE	VIP236 with NE	VIP126
786-O	Human renal cell carcinoma	188	1.1	1.2
HT29	Human colorectal cancer	245	8.7	6.8
LoVo	Human colorectal cancer	91	2.9	1.8
SW480	Human colorectal cancer	41	1.2	1.8
NCI-H292	Human lung mucoepidermoid carcinoma	209	1.8	1.5
NCI-H69	Human small cell lung cancer	486	3.0	2.9
4T1	Murine mammary carcinoma	>1000	59	49

IC_50_, half-maximal inhibitory concentration; NE, neutrophil elastase; VIP126, modified camptothecin payload.

**Table 4:** Pharmacokinetics of VIP236 and VIP126 in female NMRI nu/nu 786-O tumor-bearing mice following administration of a single i.v. dose of VIP236 (4 mg/kg) or VIP126 (1 mg/kg). The doses of VIP236 and VIP126 deliver equimolar amounts of VIP126. Pharmacokinetic parameters were estimated from mouse concentration–time profiles (*n* = 2 mice/timepoint).

PK Parameters		VIP236	VIP126
Dose	(mg/kg)	4	1
AUC_inf_	(µg·h/L)	93,400	301
CL	(L/h/kg)	0.0428	3.33
C_max_	(µg/L)	112,000	1510
V_ss_	(L/kg)	0.0688	1.54
t_1/2_	(h)	0.807	1.02

**Table 5:** Plasma and tumor pharmacokinetics of VIP236 and VIP126 in female NMRI nu/nu 786-O tumor-bearing mice following administration of a single i.v. dose of VIP236 at 4 mg/kg or VIP126 at 1 mg/kg. The doses of VIP236 and VIP126 deliver equimolar amounts of VIP126. Pharmacokinetic parameters were estimated from mouse concentration–time profiles (*n* = 2 mice/timepoint).

Compound Dosed	VIP236 (4 mg/kg)		VIP126 (1 mg/kg)
PK Parameters	VIP236	VIP126	VIP126
AUC_tumor_ (µg·h/L)	4660	318	185
AUC_plasma_ (µg·h/L)	93,400	52.1	301
AUC_tumor_/AUC_plasma_	0.0499	6.10	0.616

**Table 6:** Antitumor efficacy of VIP236 and the reference compounds in the MX-1 breast cancer, NCI-H69 lung cancer, and SW480 colorectal cancer models.

Treatment	Corresponding Payload Dose (mg/kg)	Response ^a^	Treatment/ Control Ratio ^b^
MX-1 TNBC			
VIP236, 26 mg/kg, 3 on/4 off	6.5	CR: 7/8, PR: 1/8	0.003 ***^, ###^
VIP236, 36 mg/kg, 3 on/4 off	9	CR: 6/8, PR: 2/8	0.004 ***^, ###^
VIP236, 40 mg/kg, 3 on/4 off	10	CR: 7/8, PR: 1/8	0.001 ***^, ###^
Irinotecan, 15 mg/kg, 4 on/3 off	9.5	CR: 4/8, PR: 4/8	0.002 ***
Irinotecan, 30 mg/kg, Q2/3Dx9	19	PR: 5/7, SD: 2/7	0.15 ***^, #^
Doxorubicin, 10 mg/kg, Q14Dx2	n/a	SD: 1/7, PD: 2/7	0.49 ***
NCI-H69 SCLC			
VIP236, 40 mg/kg, 3 on/4 off	10	PR: 8/8	0.06 ***^, #^
Topotecan, 0.5 mg/kg, 7 on/7 off	n/a	SD: 1/8, PD: 7/8	0.20 ***
Cisplatin, 3 mg/kg, Q3Dx14	n/a	SD: 1/8, PD: 7/8	0.25 ***
SW480 CRC			
VIP236, 26 mg/kg, 3 on/4 off	6.5	PR: 2/8, SD: 4/8, PD: 2/8	0.18 ***^, ###^
VIP236, 36 mg/kg, 3 on/4 off	9	PR: 7/8, SD: 1/8	0.09 ***^, ###^
VIP236, 40 mg/kg, 3 on/4 off	10	PR: 8/8	0.10 ***^, ###^
Irinotecan, 15 mg/kg, 4 on/3 off	9.5	PR: 5/8, SD: 3/8	0.13 ***
Irinotecan, 30 mg/kg, Q2/3D	19	PR: 4/8, SD: 3/8, PD: 1/8	0.15 ***
5-FU, 100 mg/kg, Q7D	n/a	PD: 8/8	0.54 ***

^a^ Treatment responses were determined on the last day of the vehicle group using the RECIST criteria [59]: Day 86 for MX-1, Day 31 for NCI-H69, and Day 24 for SW480. CR, complete response; PR, partial response; SD, stable disease; PD, progressive disease. ^b^ Treatment/control ratio determined on the last day of the vehicle group: Day 86 for MX-1, Day 31 for NCI-H69, and Day 24 for SW480; *** *p* < 0.001 compared with vehicle; ^#^ *p* < 0.05 compared with doxorubicin in MX-1 or cisplatin in NCI-H69; ^###^ *p* < 0.001 compared with doxorubicin in MX-1 or 5-FU in SW480. n/a, not applicable.

## Data Availability

Not applicable.

## Supplementary Materials

The following supporting information can be downloaded at: https://www.mdpi.com/article/10.3390/cancers14020391/s1, Figure S1: Cytotoxic activity of VIP126 and SN38 in parental and efflux transporter-expressing NCI-H1975 cells, Figure S2: Cytotoxic activity of VIP236 in the presence or absence of NE in various cancer cell lines.
