# Supplementary material for: A Small Molecule–Drug Conjugate (SMDC) Consisting of a Modified Camptothecin Payload Linked to an αVß3 Binder for the Treatment of Multiple Cancer Types
Source: Cancers (Basel). 2022 Jan 13;14(2):391. doi: 10.3390/cancers14020391 (PMC8773721; doi:10.3390/cancers14020391)
Supplement: Supplementary file 1 [file cancers-14-00391-s001.zip › cancers-1469458-supplementary.pdf]

Supplementary information for:

A small molecule-drug conjugate (SMDC) consisting of a modified camptothecin payload linked to an αvß3 binder for the treatment of multiple cancer types

Hans-Georg Lerchen, Beatrix Stelte-Ludwig, Charlotte Kopitz, Melanie Heroult, Dmitry Zubov, Joerg Willuda, Thomas Schlange, Antje Kahnert, Harvey Wong, Raquel Izumi and Ahmed Hamdy

Supplementary figures

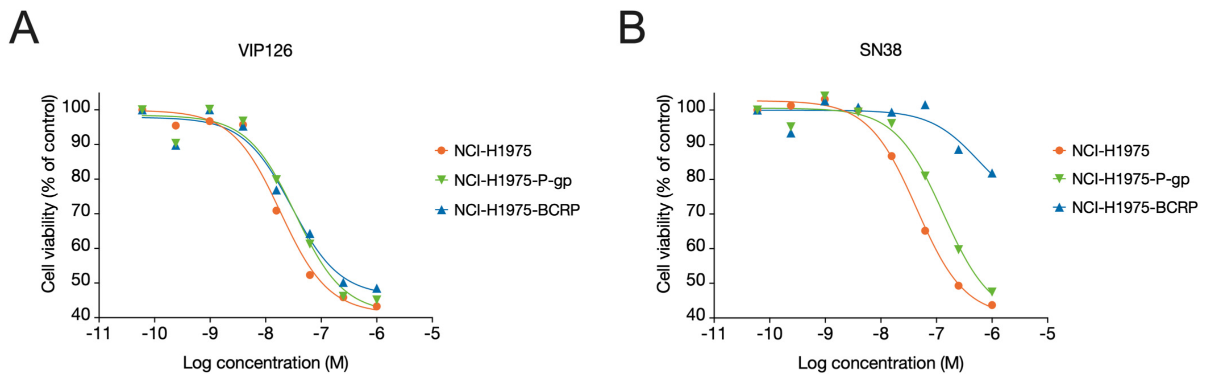

Figure S1. Cytotoxic activity of VIP126 and SN38 in parental and efflux transporter-expressing NCI-H1975 cells. The cytotoxic activity of (**A**) VIP126 and (**B**) SN38 was evaluated in parental and in P-gp or BCRP efflux transporter-expressing NCI-H1975 cells by measuring cell viability. The viability of untreated, but otherwise identically handled, cells was defined as 100%. Experiments were performed in triplicate (n = 3) in at least two independent assays.

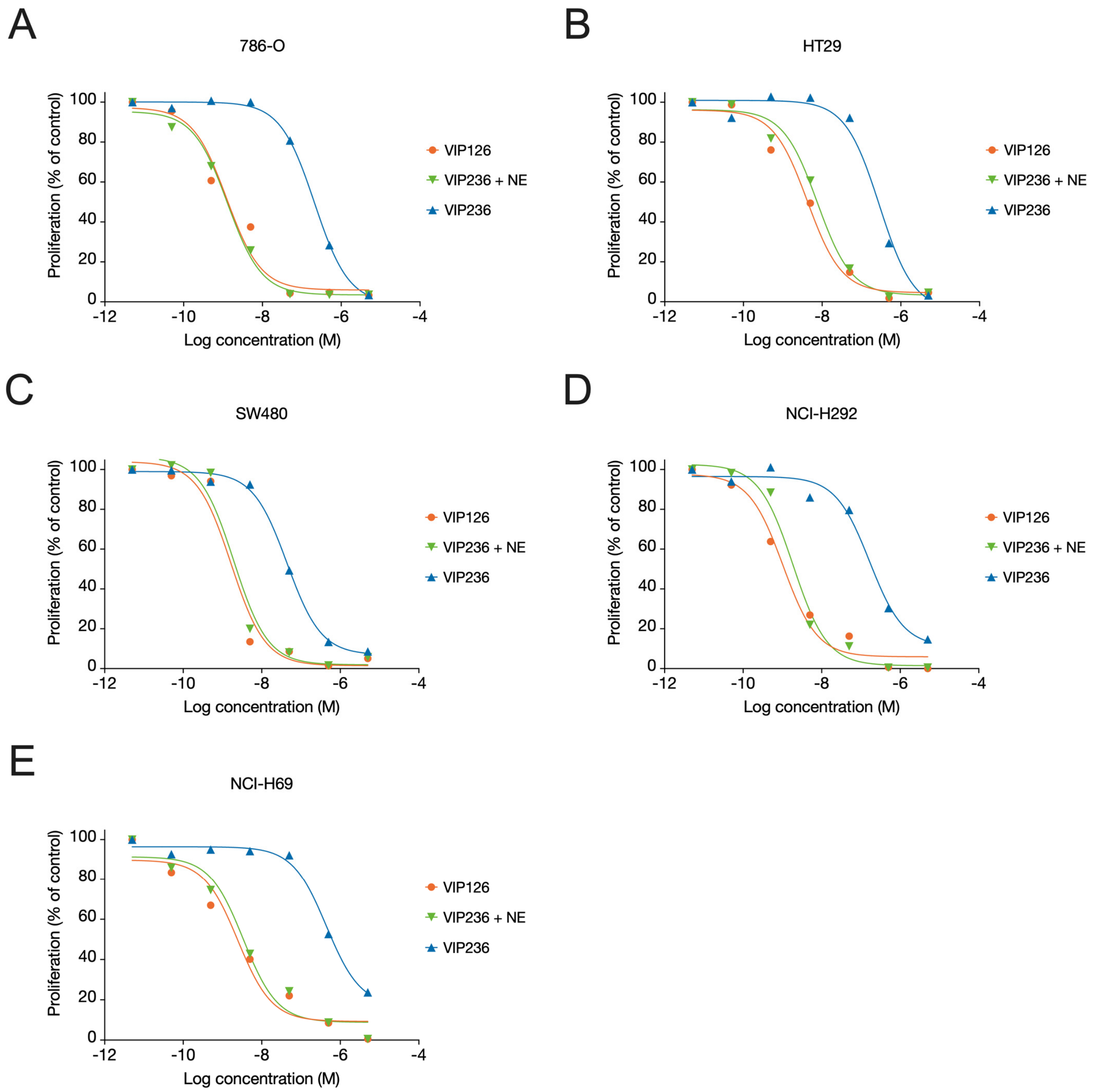

Figure S2. Cytotoxic activity of VIP236 in the presence or absence of NE in various cancer cell lines. The cytotoxic activity of the VIP236 conjugate was evaluated in the presence or absence of 10 nM NE in human (**A**) 786-O RCC, (**B**) HT29 CRC, (**C**) SW480 CRC, (**D**) NCI-H292 lung mucoepidermoid carcinoma, and (**E**) NCI-H69 SCLC cells and compared to the VIP126 payload alone. The proliferation of untreated, but otherwise identically handled, cells was defined as 100%. Experiments were performed in triplicate (n = 3) in at least two independent assays.
